# EFFECT OF A COVID-19-RELATED LOCKDOWN ON MIDDLE-AGED AND OLDER FAMILY CAREGIVERS IN SINGAPORE

**DOI:** 10.1093/geroni/igad104.2218

**Published:** 2023-12-21

**Authors:** Rahul Malhotra, Vicky Qin, Abhijit Visaria

**Affiliations:** Duke-NUS Medical School, Singapore, Singapore; Nanyang Technological University, Singapore, Singapore; Duke-NUS Medical School, Singapore, Singapore

## Abstract

Lockdowns, while limiting COVID-19 transmission, can affect care provision by family caregivers and their caregiving experience. We assessed, among 1094 family caregivers aged 50-79 years in Singapore, the (1) perceived effect of nationwide lockdown on their care provision, (2) correlates of different perceptions, and (3) association of the perceptions with negative and positive caregiving experiences. Caregivers reported whether their care provision became harder, easier, or remained the same during versus before the lockdown. Multinomial logistic regression assessed the association of caregiver, care-recipient, and caregiving-context characteristics with their perceptions. Linear regression models examined the association of their perceptions with subsequent negative and positive caregiving experiences. Care provision became harder for 36%, easier for 18% and remained the same for 46% caregivers. Care provision becoming harder (versus same) was more likely for caregivers who were male, Chinese, in worse health, caring for care-recipients with functional limitations, without caregiving support from cohabiting family before the lockdown, and with caregiving support from non-cohabiting family before the lockdown. Care provision becoming easier (versus same) was less likely among caregivers who were of higher age, unemployed, socially isolated and whose care-recipients had functional limitations. Caregivers for whom care provision became harder were worse off in negative caregiving experiences. A nationwide lockdown did not make care provision harder for all caregivers. However, those for whom it did had greater negative caregiving experiences. Heterogeneity of the effect of lockdowns and offering flexibility to non-cohabiting family who support caregiving should be considerations when implementing such disruptions.

